# The effects of the inclusion of jabuticaba peel flour on performance, metabolism, and its influence on the skin color of *Carassius auratus*

**DOI:** 10.1007/s11250-025-04845-2

**Published:** 2026-03-02

**Authors:** Rebeca Maria Sousa, Mayara Schueroff Siqueira, Marcos Paiva Scardua, Tainá Avila Pinho, Andressa C. A. B. Casari, Sandriele G. C. Deboleto, Dacley Hertes Neu, Claucia Aparecida Honorato

**Affiliations:** 1 Universidade Federal Da Grande Dourados (UFGD), Rua João Rosa Góes, 1761 - Vila Progresso, Dourados, MS 79825-070 Brasil; 2 Universidade Federal de Mato Grosso do Sul (UFMS), Av. Costa e Silva, s/n - Pioneiros, Campo Grande, MS 79070-900 Brasil

**Keywords:** Blood parameters, Goldfish, Metabolic enzymes, Ornamental fish

## Abstract

This work aimed to evaluate the inclusion of jabuticaba peel flour (JPF) in skin color and productive performance, biochemical, blood, and enzymatic parameters of *Carassius auratus*. 72 fish (weight 9.67 ± 0.37 g, length 7.38 ± 0.18 cm) were distributed into four treatments (0.00; 1.00; 1.50 and 2.00% JPF), in a completely randomized design with three replications with six fish per box. After 60 days, to evaluate the performance of the fish. To assess the skin color of the fish, a portable photo colorimeter was used using the Hunter coordinate system, where the coordinates of L*, a*, and b*. Blood samples were taken for biochemical analysis regarding glucose, triglycerides, and cholesterol. The analysis of ALT and AST, albumin and triglycerides in liver tissue, and amylase, lipase, nonspecific protease, and alkaline phosphatase in the intestine. Results obtained showed that there was a significant difference about feed intake and specific growth rate with the inclusion of JPF. It was observed that fish fed with 1.00 and 1.50% JPF obtained an increase in brightness represented by the *L* coordinate and shades of yellow represented by the b coordinate. Glucose increased with 2.00% of JPF. AST activity was higher with 1.00% JPF and ALT was reduced with the inclusion of JPF, demonstrating an ability to protect against possible liver damage. The inclusion of JPF is effective at levels of up to 1.5% to increase the brightness of the skin of *Carassius auratus*.

## Introduction

As a growing market, ornamental fish farming is represented by the pet market occupying the fourth place when it comes to companion animals, becoming an important commercial activity, in addition to one of the most popular hobbies in the world (Villar-Martinez et al. 2013). The growing interest in this market, the low demand for the activity, and the high value of ornamental fish make special attention necessary, demanding that producers offer a differentiated “product” aiming at bright colors, format and originality in the species of commercial value.

Maintaining these fish always with vigor and intense color until they reach the final consumer has been one of the main obstacles faced by the ornamental fish producer due to the handling and harvesting processes, as the fish go through periods of stress, which can cause the decrease of this vigor referring to the color of these fish (Nascimento et al. 2019). One of the ways to maintain and optimize the color of these fish species is to add pigments to their diet (Chatzifotis et al. 2005; Maiti et al. 2017), thus creating a demand for food colors from natural sources (Lopes et al. 2007; Ndong and Fall 2011; Eaton et al. 2016; Cal et al. 2017).

Pigments are chemical compounds that absorb light, producing color by specific cells (chromatophores), which by different processes recognized by the brain reflect color (Delgado-Vargas et al. 2000). Therefore, natural pigments are being studied to improve the color intensity in ornamental fish, since color diversity, in addition to playing an important role in the biological activities of many organisms, represents one of the main factors when choosing fish (Dananjaya et al. 2017; Jorjani et al. 2018).

The use of bioactive substances has received increasing attention due to increased feed palatability followed by increased growth (Takoaka et al. 2011) and an improvement indigestive enzyme activity (Rashidian et al. 2020) and, along with other compounds act to maintain and intensification of the skin color of these animals (Lopes et al. 2007). In this context, there is a demand for specific diets for ornamental fish, which can use bioactive substances that keep them colorful and showy, generating a growing interest in research aimed at the use of these substances, which can be extracted from foods and their by-products (shell, seeds and bagasse) from the Brazilian fauna, which can be incorporated into other foods and effectively improve the nutritional value added and improve the quality of life of fish (Aziz et al. 2012; Morales et al. 2016).

Jabuticaba (*Plinia cauliflora*), is a Brazilian fruit that contains phenolic compounds, such as anthocyanins, in its composition, which can contribute to improving the skin pigmentation of fish (Leite-Legatti et al. 2012). The jabuticaba peel represents 50% of the fruit, a residue rich in significant amounts of bioactive compounds, which can act as a nutritional additive in the incorporation in animal feeds, therefore, it can act as a natural dye (Ferreira et al. 2012; Morales et al. 2016; Marquetti et al. 2018). In addition, the use of nutritional additives that have bioactive promoting health and well-being benefits, in addition to intensifying the skin color fish (Eaton et al. 2016) and in the hepatoprotective action (Leite et al. 2011).

Ornamental fish have a variety of color and shape combinations, and among these ornamental fish the Carassius auratus is a freshwater fish, is closely related to the common carp (*Cyprinus carpio*) and grass (*Ctenopharyngodon idella*). It has great commercial value-added due to its great ability to assume different forms (polymorphism), as well as varied coloration, allowing great variations within the same species (Protas and Patel 2008; Kumar et al. 2017). In view of this, this study aimed to evaluate the inclusion of bioactive from jabuticaba peel flour in the production performance, biochemical, blood and enzymatic parameters and their influence on the skin color of *Carassius auratus*.

## Materials and methods

### Ethics statement

The procedures for experimenting followed the procedures approved by the Animal Use Ethics Committee (CEUA/UFGD) of the Federal University of Grande Dourados, MS, Brazil under protocol No. 34/2017.

### Characterization of raw material

The jabuticaba variety Sabará (*Myrciaria jabuticaba* (Vell.) Berg.), purchased in the municipality of Dourados (Mato Grosso do Sul), was used, which were washed and sanitized and pulped manually. Fresh jabuticaba bark samples were dried at 70 °C in a convective dryer for further grinding.

The approximate composition of the jabuticaba peel flour was performed following the analytical procedures described by AOAC (2005). Soluble solids (SS) were determined by direct reading in an ATTO-2WAJ digital refractometer and the results were expressed in degrees Brix. Titratable acidity (TA) was determined by neutralizing the solution with 0.1 N sodium hydroxide (AOAC 1996), with the results expressed as a percentage of citric acid. To determine the total phenolic components, the Folin-Ciocalteu (1927) reagent was used. The anthocyanin content was evaluated according to Lees and Francis (1972). The determination of the antioxidant activity (AA) was carried out by means of DPPH free radical reducing capacity (1,1-diphenyl-2-picrylhydrazyl); and ABTS free radical capture (2,22-azinobis acid (3-ethylbenzothiazoline-6-sulfonic acid), according to Rufino et al. (2007a, b).

The composition of jabuticaba peel flour has: Moisture 8.62 ± 0.09 g 100 g^− 1^; Ash 3.28 ± 0.06 g 100 g^− 1^; Proteins 7.66 ± 0.29 g 100 g^− 1^; Ether extract 3.83 ± 0.21 g 100 g^− 1^; Crude fiber 61.18 ± 0.26 g 100 g^− 1^; Total carbohydrate 77.15 ± 0.48 g 100 g^− 1^; Soluble solids 3.17 ± 0.15° Brix; Total caloric value 373.73 ± 1.23 kcal 100 g^−^1; Total anthocyanins 20.44 mg 100 g^− 1^, Phenolic compounds 4.84 mg of EAG 100 g sample^− 1^. The antioxidant capacity by the free radical capture method ABTS 257.25 ± 7.02 µM Trolox (g sample^− 1^), and by the DPPH method (0.61 ± 0.01 g fruit (g DPPH)).

### Experimental diets

Extruded commercial feed containing 40% crude protein was used as a base for the incorporation of jabuticaba peel flour. The ration was crushed until it turned into powder, then the jabuticaba peel flour moistened with water (approximately 30%) was added, followed by extrusion. Extrusion was carried out manually in a meat grinder with a 5 mm matrix plate. Afterward, the obtained pellets were exposed in trays for drying in a natural environment for 24 h. Pellets were stored in covered glass containers under refrigeration (-18 °C) until use.

Three diets were prepared to contain: 1.00; 1.50 and 2.00% jabuticaba peel flour and a control diet (no addition of jabuticaba peel flour). Diets were evaluated for the chemical composition according to the methodology described by AOAC (2005) (Table 1).

**Table 1:** Ingredients and approximate composition of the diets

Ingredients	Inclusion levels of Jaboticaba peel flour (%)			
	0.00	1.00	1.50	2.00
^1^Commercial Diet	100	990	985	980
^2^JPF (g)	-	10	15	20
^**3**^ **Calculated composition**				
Moisture	9.6	9.6	9.6	9.6
Crude protein	40.9	40.6	40.7	40.3
Ethereal extract	10.3	10.2	10.2	10.1
Mineral matter	10.5	10.5	10.5	10.4
Fibrous matter	2.5	2.5	2.7	2.5
Calcium	1.4	1.4	1.4	1.4
Phosphor	0.8	0.8	0.8	0.8
^4^NNE	24.0	24.5	24.1	24.9

^1^Commercial feed basic ingredients (g.kg^− 1^): Meat and bone meal; Blood meal; Wheat bran; Integral corn; Rice wax; Marine fish oil; Bird oil; Hemoglobin; Flour of viscera and soybean meal. Data represented with mean and standard deviation. The number of samples. *n* = 3. ^2^JPF: jaboticaba peel flour; ^3^The analyzes were carried out in the UNIGRAN soil laboratory. ^4^NNE: non-nitrogenous extract. 08997659.2012.711266

### Experimental trial

Seventy-two specimens of *C. auratus* were used with an average body weight of 9.67 ± 0.37 g and total length of 7.38 ± 0.18 cm, distributed in 12 boxes (60 L) with six fish per box. Those used in fish feeding consisted of diets with the addition of jabuticaba peel flour (control – without the addition of JPF; 1.00; 1.50 and 2.00%) in a completely randomized design with four treatments and three replications in a system of water recirculation. The boxes were kept under natural photoperiod (12 h of light: 12 h of dark).

Fish were fed twice a day at a rate of approximately 10% of live weight. After 60 days, the data related to growth performance (body weight and length) were measured: Weight gain = final weight-initial weight; SGR = ((ln of final weight - ln of initial weight) × 100) /days of experiment; FC = feed intake / gain in weight; Survival = (final number of fish / initial number of fish) × 100.

### Fish skin pigmentation

The assess fish skin pigmentation, six fish from each treatment were used. These fish were placed on a smooth white surface and sampled in the dorsal region, just below the dorsal fin, in the fish in vivo.

The skin color gain of the fish was performed using a portable colorimeter Chroma Meter CR-400 (Konica Minolta^®^), using the Hunter L*, a*, b* coordinate system, which measured the intensity of L* representing brightness or luminosity (-100, black and + 100, white), the chromaticity of the a*, represented by shades of green (-100) and red (+ 100) and chromaticity of b*, represented by shades blue (-100) and yellow (+ 100) (Rezende et al. 2012). The color gain results include data referring to the averages obtained at 20, 40 and 60 days of the experiment.

### Biochemical analyses

For blood tests, samples were obtained by veno-caudal puncture of three fish from each replicate using 3 mL heparinized syringes for glucose, triglycerides and cholesterol analyses. The tests were performed in portable equipment corresponding to each parameter, following the protocol of the manufacturer Accutrend^®^ (Cobas). After blood collection, the animals were anesthetized on ice sacrificed to remove liver and intestine samples. All samples were stored at -80 °C until further analysis.

Samples of the 100 mg of liver were homogenized with sodium phosphatase buffer ((glycerol v/v in 20 mM sodium phosphate buffer and 10 mM Tris - pH 7.0) in a Potter-Elvehjem type homogenizer, then the samples were centrifuged for five minutes at 3000 rpm. The supernatant was used for assays of albumin and triglyceride activity and the enzymes alanine aminotransferase (ALT), aspartate aminotransferase (AST), which were determined by a modification of the method of Reitman and Frankel (1957). The determination of the parameters of metabolic and digestive enzymes was obtained with kits from Gold Analisa Diagnóstica^®^ specific for these enzymes. Sample readings were performed by spectrophotometer (BIO PLUS S 200 semi-automatic spectrophotometer), with the light of the appropriate wavelength for each test.

For the assays of digestive enzymes amylase, lipase, nonspecific protease, and alkaline phosphatase. 100 mg of intensive were used and homogenized with sodium phosphate buffer (glycerol v/v in 20 mM sodium phosphate buffer and 10 mM Tris - pH 7.0) in a Potter-Elvehjem type homogenizer. Subsequently, centrifuged for five minutes at 3000 rpm. Sample readings were performed in triplicate by spectrophotometry (Bioplus S-200 semi-automatic spectrophotometer), with the light of the appropriate wavelength for each test.

### Statistical analyses

Data were submitted to the Shapiro-Wilk normality test. Then, ANOVA was performed. When significant differences were observed, the means were compared using the Tukey test. For color, data were analyzed using the polynomial regression test with treatment and time as categorical variables, at a significance level of 5%. Data are expressed as mean and SD.

## Results

There was no mortality in the treatments tested. During the experimental period, the average temperature remained at 26.5 °C. dissolved oxygen averaged 5.11 mg/L, remaining within optimal conditions. The average pH was 7.16 and total ammonia remained at an average of 0.3 mg/L.

The addition of jabuticaba peel flour (JPF) promoted favorable changes in growth revealed in the results of the specific growth rate (SGR) and total consumption (Table 2).

**Table 2:** Growth performance parameters of Carassius auratus fed with different levels of Jaboticaba Peel flour for 60 days

Variables	Inclusion levels of Jaboticaba peel flour (%)			
	0.00	1.00	1.50	2.00
Final weight (g)	19.51 ± 0.90	20.38 ± 0.82	20.50 ± 0.64	20.88 ± 0.39
Weight gain (g)	9.63 ± 0.83	10.67 ± 0.52	11.03 ± 0.37	11.17 ± 0.26
Standard length (cm)	5.72 ± 0.14	5.59 ± 0.24	5.58 ± 0.14	5.53 ± 0.20
Total length (cm)	9.23 ± 0.15	9.14 ± 0.16	9.09 ± 0.19	8.97 ± 0.34
Body Height (cm)	3.68 ± 0.03	3.76 ± 0.08	3.69 ± 0.03	3.74 ± 0.14
Total consumption (g)	141.16 ± 5.22c	187.16 ± 5.20a	166.03 ± 4.15b	168.12 ± 5.37b
^2^FC (g/g)	1.52 ± 0.19	1.65 ± 0.17	1.35 ± 0.07	1.34 ± 0.02
^3^SGR (%)	0.17 ± 0.01b	0.18 ± 0.01ab	0.19 ± 0.01a	0.19 ± 0.01a
Survival (%)	100	100	100	100

Distinguished letters report a statistical difference (*P* < 0.05) by the Tukey test. ^2^FC = feed conversion; ^3^SGR = specific growth rate. Values expressed as mean and ± SD

The development of *C. auratus* improved when JPF was included in their diet, with an increase in total feed consumption promoted by the inclusion of JPF, demonstrating the acceptability and palatability of the product regardless of the level of inclusion. The specific growth rate (SGR) proved to be more efficient with the inclusion of 1.00 and 1.50% JPF.

Biochemical parameters are shown in Table 3, the triglyceride values did not differ, however, the inclusion of 2.00% JPF increased circulating glucose in *C. auratus*. The results for digestive values, the activity of the liver and metabolic enzymes with different levels of inclusion of flour from jabuticaba peel are shown in Table 3. Regarding digestive enzymes, a significant difference (*P* > 0.05) was observed regarding lipase in groups with 1.00 and 2.00% inclusion of JPF. Amylase, Protease and Alkaline Phosphatase showed no difference with the variation of the diet.

**Table 3:** Biochemical parameters an enzymatic of *Carassius auratus*, the activity of digestive enzymes, and liver metabolic enzymes fed with different levels of Jaboticaba Peel flour for 60 days

Variables	Inclusion levels of Jaboticaba peel flour (%)			
	0.00	1.00	1.50	2.00
**Biochemical parameters (mg/dL)**				
Glucose	45.11 ± 1.34^b^	41.66 ± 8.05^b^	48.43 ± 6.53^b^	78.89 ± 4.09ª
Triglycerides	70.00 ± 10.8	75.66 ± 8.01	80.00 ± 14.14	91.66 ± 26.07
Cholesterol	254.33 ± 23.57	258.66 ± 29.22	205.33 ± 15.79	223.88 ± 14.46
**Liver metabolic enzymes (U/mg)**				
^1^ALT	29.78 ± 10.26^a^	20.45 ± 3.00^ab^	21.78 ± 4.52^ab^	10.89 ± 1.10^b^
^2^AST	121.33 ± 14.11^b^	120.00 ± 37. 33^a^	68.11 ± 39.95^ab^	102.53 ± 18.78^ab^
AST: ALT^1^	0.91 ± 0.59	6.12 ± 1.92	4.52 ± 3.01	6.46 ± 4.57
**Liver tissue biochemical parameters (U/mg)**				
Albumin	0.14 ± 0.05	0.29 ± 0.13	0.27 ± 0.02	0.15 ± 0.03
Triglicerídeos	88.66 ± 29.29^b^	94.00 ± 13.63^ab^	150.66 ± 13.10^ab^	109.94 ± 9.03^a^
**Digestive enzymes (U/mg)**				
Amylase	1.00 ± 0.02	1.04 ± 0.08	1.00 ± 0.02	1.05 ± 0.04
Lipase	178.33 ± 76.88^b^	312 ± 47.8^a^	165 ± 45.8^b^	207 ± 43.8^ab^
^3^Nonsp. Protease	2.14 ± 1.67	1.31 ± 0.54	0.98 ± 0.48	1.68 ± 1.01
^4^Alkaline phosph.	1098.33 ± 374.63	1092.55 ± 535.56	1253.11 ± 446.87	1161.67 ± 379.93

Different letters report statistical differences (*P* < 0.05) by Tukey’s test. ^1^ALT: Alanine aminotransferase. ^2^AST: Aspartate aminotransferase. ^3^Unspecific Protease; ^4^Alkaline Phosphatase. Values are expressed as mean and standard deviation

AST activity was higher in fish fed a diet containing 1.00% JPF (Table 3); ALT showed a reduction in its activity due to the variation in the inclusion of JPF when compared to the control group. The levels of hepatic triglycerides showed a difference (*P* > 0.05) with the increase the inclusion of JPF. Albumin levels showed no significant difference, but there was an increase with the inclusion of JPF when compared to the control. In our study, we chose to investigate the activity of ALT and AST in the liver and not in the blood in order to obtain more concrete results on the effect of jabuticaba peel flour on the liver.

**Fig. 1:**
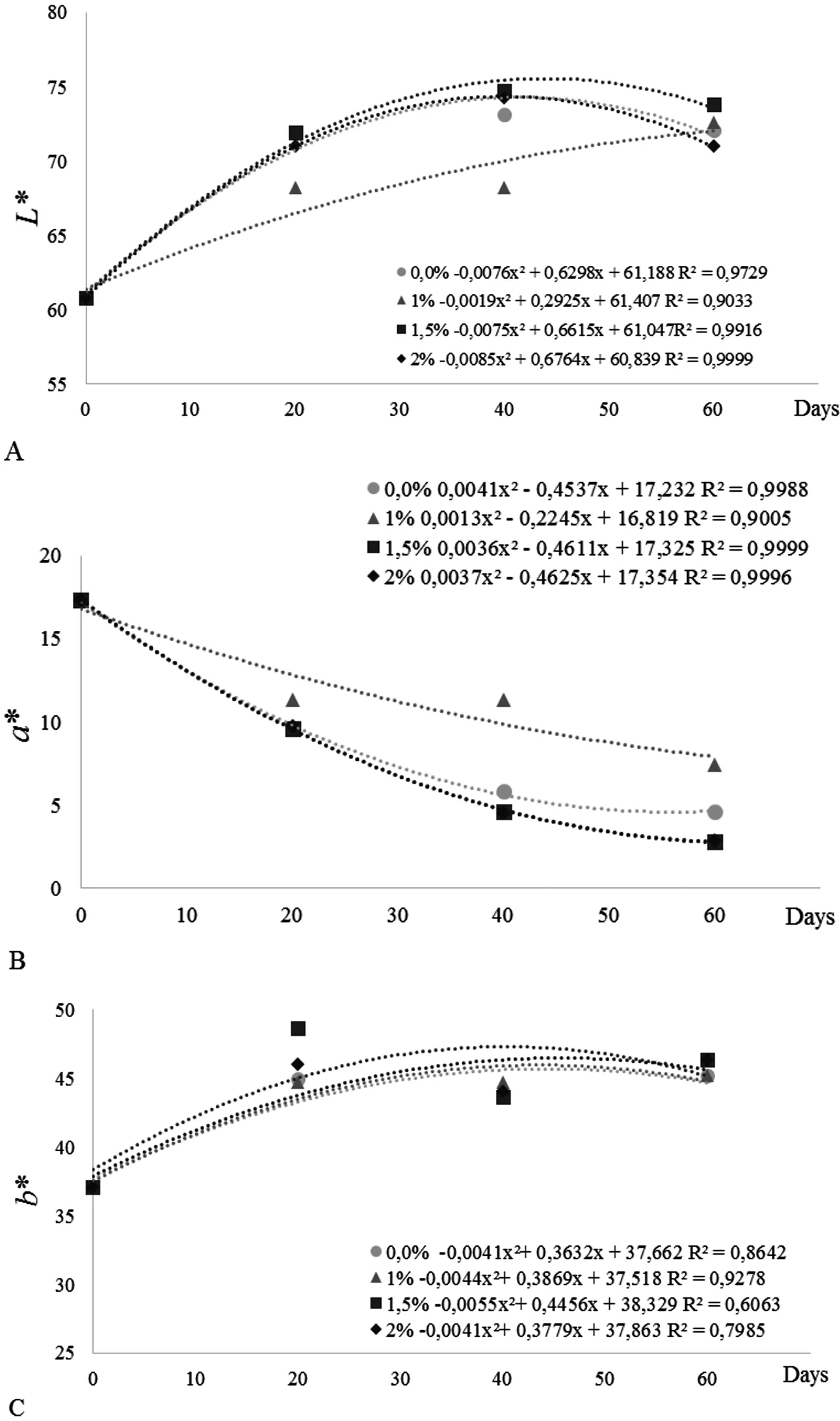
Color parameters L* (**A**), a* (**B**), b* (**C**) referring to *Carassius auratus* skin pigmentation fed with different levels of jaboticaba peel flour (JPF) for 60 days

Brightness and yellow chromaticity (b*) of fish fed diets with the addition of JPF were fitted to a quadratic equation (Fig. 1A and C). The addition of JPF intensifies L* and b* when 1.50% JPF was added to the diet. Regression analysis showed that the relationship between L* and b* parameters responds to the moment when the fish are receiving food. However, increase the level of JPF in the diet resulted in a reduction in red (a*) (Fig. 1B). In this study, it was observed that the inclusion of JPF intensifies the luminosity (L*) and yellow hue (b*) of *C. auratus* when 1.50% JPF was included in the diet.

**Fig. 2:**
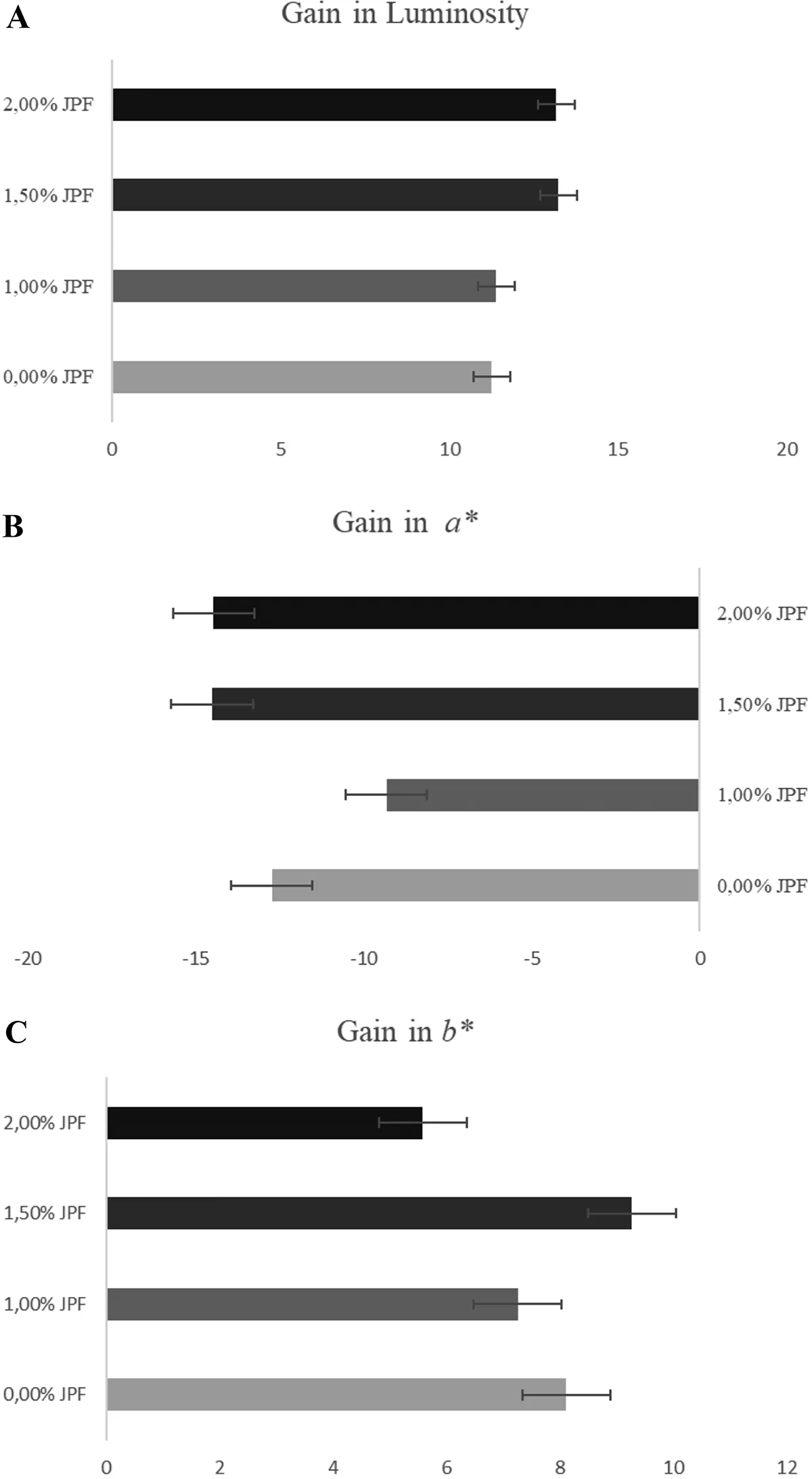
The luminosity gain (**A**), chromaticity a* (**B**), and b* (**C**) in the skin of *Carassius auratus* fed with jaboticaba peel flour (JPF)

The results of the polynomial regression analysis showed that 1.5% of JPF inclusion is more efficient for both brightness gain and yellow hue gain, in which luminance gain up to 44 days, and for yellow hue for inclusion of JPF is more efficient up to 40 days of feeding. The loss of red color was observed due to the decrease in a* chromaticity in all treatments, however, in fish fed with 1.00% JPF the loss of this color was smaller, indicating that the JPF response to red pigmentation is related to the low percentage of inclusion in the diet (Fig. 2B). The best color gain rate was obtained in the group of fish fed 1.50% JPF for brightness and yellow hue (b*) (Fig. 2A and B).

## Discussion

In this study, we observed that the inclusion of JPF promotes the growth of *C. auratus*, as well as studies carried out with the inclusion of pomegranate (*Punica granatum* L.) bioactive (Kumar et al. 2017), Hibiscus (*Hibiscus rosasinensis*) (Sinha and Asimi 2007); Annatto (*Bixa orellana*) (Fries et al. 2014; Danajaya et al., 2017), African tulip flower (*Spathodea campanulata*), red paprika (*Capsicum annuum*) (Kumar et al. 2017). This is because bioactive products have properties that have the ability to modulate one or more metabolic processes, promoting better health conditions (Angiolillo et al. 2015).

Changes in serum biochemical parameters may be considered an index of fish health when studies are carried out with manipulations in their diets (Yılmaz and Ergun 2012; Rashidian et al. 2020).

The decrease in ALT, reveals the hepatoprotective effect of anthocyanins present in JPF. Changes in AST activities are considered insignificant in this situation where the values below those of reference for these enzymes are observed (compared to the control group). These results are corroborated by production of albumin that is synthesized exclusively in the liver, which has increased activity compared to the control group, ensuring the hepatoprotective effect (Silva et al. 2011). The action of plants as hepatoprotective with decreased ALT and AST activity is reported in fish (Fuentes-Quesada et al. 2018; Ota et al. 2019). Increased activity of these enzymes in the liver (Aich et al. 2011; Humtsoe et al. 2007) as well in the blood (Abdelhiee et al. 2021; Gonçalves et al. 2018) are indicators of damage to hepatocytes.

Digestive activity depends, to some extent, on the amount of food ingested and its specific substrate (Dorce et al. 2020). This study of the inclusion of JPF did not obtain protease responsiveness since there was no increase in the specific substrate for this enzyme. The foregut is the most important site for lipid digestion (De Almeida et al. 2006), a source of great importance for fish nutrition. The change in digestive lipase activity may be indicative of an improvement in digestion rates compared to the use of nutritional additives.

The bioactive compounds present in the jabuticaba peel flour, are responsible for the color that varies from bright red to violet and from white to light yellow (Bobbio and Bobbio 2003; Lima et al. 2011). The chromaticity of a* decreased when there was the inclusion of JPF, with no gain related to the red tone in the fish skin. This can be explained by the fact that the rate of deposition of pigment present in the diet varies with the fish ability to convert it (Fries et al. 2014).

Du et al. (2018) point out that fish color diversity is cream, and color patterns can vary between species, as well as between populations of a species (sexual dimorphism, polychromatism and geographic variation), as well as within of individuals. Coloration is a relevant characteristic of many animals, which help in adapt to the environment, in addition to playing a significant role in social behavior, camouflage and mating (Protas and Patel 2008; Rodgers et al. 2010; Maan and Sefc, 2013). However, fish do not have the ability to synthesize the pigments that form the color, and they need supplementation in the diet.

Ectothermic vertebrate animals lack the ability to synthesize pheomelanin, a type of melanin produced by chromatophores (pigment cells) responsible for red or yellow pigmentation. This pigmentation occurs through a process called melanogenesis, which is produced from the amino acid tyrosine, a process that begins with the oxidation of L-tyrosine, going through a series of transformations, generating pheomelanin (Grampel and Visconti 2014; Cal et al. 2017). Observing the values obtained in relation to the chromaticity of a* it can be suggested that there is a possible deficiency of tyrosine in the jabuticaba peel flour, since the yellow and red tones are directly influenced by the origin of the pigment in the diet (Kaur and Shah 2017), it is suggested that in future studies with the use of JPF in the feeding of *C. auratus*, a profile of amino acids present in the flour and feed should is made.

Thus, we conclude that the inclusion of jabuticaba peel flour is efficient as a food additive, with the most effective concentration being 1.5% of inclusion. Carassius auratus fed with jabuticaba peel flour will have better growth and greater skin luminosity without negatively affecting liver function.

## Data Availability

The data that support the findings of this study are available from the corresponding author, upon reasonable request.
